# Correlation between vegetation and environment at different levels in an arid, mountainous region of China

**DOI:** 10.1002/ece3.3088

**Published:** 2017-06-14

**Authors:** Nannan Gao, Jihua Zhou, Xiaolong Zhang, Wentao Cai, Tianyu Guan, Lianhe Jiang, Hui Du, Dawen Yang, Zhentao Cong, Yuanrun Zheng

**Affiliations:** ^1^ Key Laboratory of Resource Plants Beijing Botanical Garden West China Subalpine Botanical Garden Institute of Botany Chinese Academy of Sciences Beijing China; ^2^ University of Chinese Academy of Sciences Beijing China; ^3^ State Key Laboratory of Hydro‐science and Engineering Department of Hydraulic Engineering Tsinghua University Beijing China

**Keywords:** environmental factors, landscape metrics, mountainous region, vegetation pattern

## Abstract

Vegetation patterns and spatial organization are influenced by the changing environmental conditions and human activities. However, the effect of environment on vegetation at different vegetation classification levels has been unclear. We conducted an analysis to explore the relationship between environment and vegetation in the land use/land cover (LULC), vegetation group, vegetation type, and formation and subformation levels using redundancy analysis with seven landscape metrics and 33 environmental factors in the upper reaches of the Heihe River basin in an arid area of China to clarify this uncertainty. Atmospheric counter radiation was the most important factor at the four levels. The effect of soil was the second determinant factor at three levels (except in vegetation formation and subformation level). The number of variables whose relationship to vegetation reached significant levels varied from 26 to 28, and 20 variables were the same at all four levels. The factors affecting vegetation were basically the same at vegetation group level and vegetation‐type level. It was sufficient to analyze the relationship between environmental and vegetation patterns only in LULC, vegetation group and vegetation formation and subformation level in mountainous regions; different factors should be considered at different vegetation levels.

## 1 INTRODUCTION

The appropriate interpretation of the relationship between vegetation types and environment (climate) is one of the main tasks of plant ecologists (Manley, 1961; Motzkin, Wilson, Foster, & Allen, 1999). Understanding the vegetation–environment relationship is essential, especially for improving knowledge of the effect of global change on ecosystems and the feedback of ecosystem to climate (Arneth, 2015; Austin, 2013; Huete, 2016). Understanding on responses of vegetation to climate change could improve predictions of the future consequences of climate change on ecosystems, biodiversity, and our own food security and welfare (Huete, 2016). The vegetation–environment relationship also can quantitatively disclose the interactions between driving factors and environmental processes and patterns, and thus help identify the main factors leading to environment changes (Sohoulande Djebou, Singh, & Frauenfeld, 2015).

This relationship can be analyzed by models (Graumlich & Davis, 1993; Yang et al., 2016; Zuo et al., 2012), metrics (Peng, Mi, Qing, & Xue, 2016; Schindler, Von Wehrden, Poirazidis, Wrbka, & Kati, 2013; Sohoulande Djebou et al., 2015; Zhang, Van Coillie, De Clercq, Ou, & De Wulf, 2013), or statistical calculation (Carleton, 1984; Dias & Melo, 2010). Landscape metrics is often used to quantify vegetation pattern and spatial organization and is sensitive to scale, as are many other ecological approaches (Bekker, Clark, & Jackson, 2009; Gardner, Milne, Turnei, & O'neill, 1987; Meentemeyer & Box, 1987; Turner, O'neill, Gardner, & Milne, 1989). However, they are usually carried out at one level, mostly at the land use/land cover (LULC) level, and so are inadequate to illuminate the vegetation–environment relationship (Hejcmanovā‐Nežerková & Hejcman, 2006; Peng et al., 2016). Other research focused on fractal dimensions and their relationship with environmental factors that varied between plant and landscape with a focus on phytoecology (Burrough, 1981). The problem with this method is that fractal dimensions can just indicate one aspect of vegetation patterns. There is, therefore, a big gap in the systematic description of the vegetation–environment relationship (Burrough, 1981).

Vegetation is influenced by various ecological factors (e.g., precipitation, temperature, light, soil and site conditions) and human disturbance, such as cultivation activities, road traffic, and urban land use. Landscape metrics are also shaped by topographical determinants, such as altitude, aspect, and slope (Geri, Rocchini, & Chiarucci, 2010). Therefore, investigating vegetation response to environment at multiscales instead of only at the LULC scale is necessary to meet different needs in resource management (Peng et al., 2016).

The analysis of some studies on the relation of soil and vegetation, precipitation and vegetation, elevation and vegetation, and environment and vegetation was based on a few selected factors, such as choosing mean annual precipitation to represent the factor “water” that may impact vegetation. However, the correlation between mean annual precipitation and vegetation may be not significant; alternatively, monthly average precipitation during the growing season or monthly evapotranspiration might be a much better way to analyze the effect of water on vegetation. It is reasonable to take as many environmental factors as possible into account, especially in highly heterogeneous environments and vegetation (Cheng et al., 2014).

The Qilian Mountains in north‐western China are located in the ecotone of the Qinghai–Tibet Plateau, the Loess Plateau and the Central Asian desert (Chen, Peng, Huang, & Lu, 1994). The region's vegetation is typical of alpine vegetation in an arid area, and it is an ideal site for understanding the relationship between vegetation and a highly heterogeneous environment (Cheng et al., 2014). However, there has been no attempt to systematically understand the vegetation–environment relationship at different scales (Cheng et al., 2014).

In this study, we used a direct ordination approach to understand correlations between environmental factors and vegetation metrics in a highly heterogeneous environment at different scales and to find the differences in these correlations at different scales and their applications in resource management.

## 2 MATERIALS AND METHODS

### 2.1 Site description

**Figure 1:**
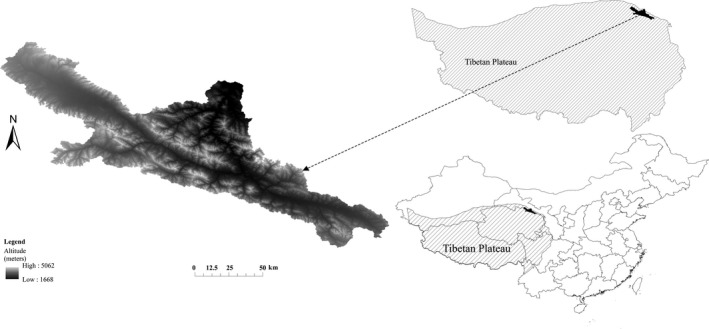
Location of study area

The upper reaches of the Heihe River basin are located in the far northeastern portion of the Tibet Plateau in central China (Figure 1). The Heihe River is the second largest inland river of China and flows through Qinghai and Gansu provinces and the Inner Mongolia Autonomous region. The basin is a typical arid watershed bounded between longitudes 98.56°E and 101.16°E and latitudes 37.63°N and 39.15°N.

The upper reaches of the Heihe River basin is the runoff yield area for the entire watershed and covers an area of about 10,009 km^2^. The annual precipitation varies in the range 149–486 mm with more than 60% concentrated in the summer. The mean annual temperature (MAT) ranges from 6.9 to −9.8°C, with cooler averages at increasing elevation (climate data sourced from WorldClim, http://www.worldclim.org/). Precipitation increases from west to east and north to south in the study area, with temperature having the opposite trend (Gao, Hrachowitz, Fenicia, Gharari, & Savenije, 2014; Qin et al., 2013; Zhao, Nan, & Cheng, 2005).

### 2.2 Vegetation and environmental data

**Figure 2:**
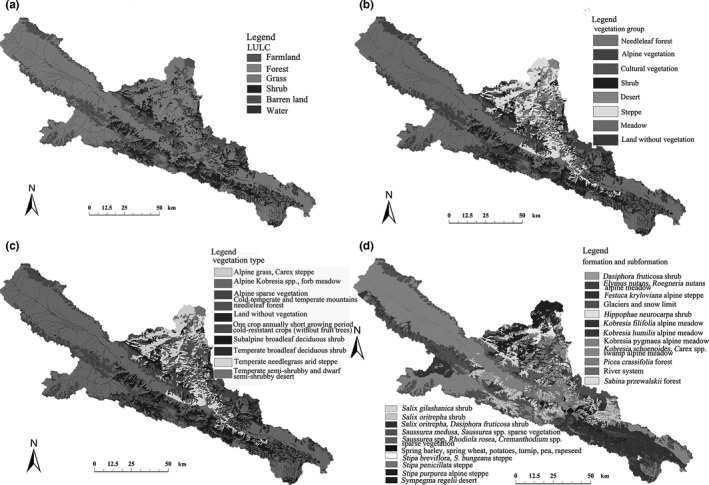
Vegetation classifications at four levels. The levels were land use/land cover (LULC) (a), vegetation groups (b), vegetation types (c), and vegetation formation and subformation (d)

The vegetation data were modified and improved from the digital Vegetation Map of the People's Republic of China (1:1,000,000) (Editorial Committee of Vegetation Map of China, CAS, 2007) with remote sensing and field data and were available on the website maintained by the Cold and Arid Regions Sciences Data Center at Lanzhou (http://westdc.westgis.ac.cn, https://doi.org/10.3972/heihe.426.2014.db). This region contains seven vegetation groups, nine vegetation types, and nineteen formations and subformations (Table 1 and Figure 2). The lowlands (1,600–2,400 m) are mainly desert, and the upper regions (2,400–2,800 m) are steppe consisting of *Stipa* spp., with needle‐leaf forest in the north ranging from 2,400 to 3,200 m, shrub‐meadows from 3,200 to 4,000 m and alpine vegetation, mainly *Saussurea* spp., in areas higher than 4,000 m. Glaciers form at the peaks of some mountains. The main land use is pasture; the forest is protected by the government; and logging has been forbidden in recent years. Some cultivated vegetation is grown near county towns, but cultivation covers an area of less than 1% in this region.

**Table 1:** Categories used to show the vegetation distribution in the study area

Coarse			Fine
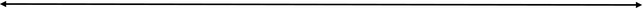			
LULC	Vegetation groups	Vegetation types	Vegetation formations and subformations
Farmland (1%)	Cultural vegetation (1%)	One crop annually short growing period cold‐resistant crops (without fruit trees) (1%)	Spring barley, spring wheat, potatoes, turnip, pea, rapeseed (1%)
Forest (5%)	Needleleaf forest (5%)	Cold‐temperate and temperate mountains needleleaf forest (5%)	*Picea crassifolia* forest (5%)
Pasture (55%)	Steppe (12%)	Temperate needlegrass arid steppe (6%)	*Festuca kryloviana* alpine steppe (3%) *Stipa penicillata* steppe (2%) *Stipa breviflora*,* S. bungeana* steppe (1%)
		Alpine grass, *Carex* steppe (6%)	*Stipa purpurea* alpine steppe (6%)
	Meadow (43%)	Alpine *Kobresia* spp. forb meadow (43%)	*Kobresia humilis* alpine meadow (3%) *Kobresia pygmaea* alpine meadow (26%) *Elymus nutans*,* Roegneria nutans* alpine meadow (1%) *Kobresia filifolia* alpine meadow (8%) *Kobresia schoenoides*,* Carex* spp. swamp alpine meadow (5%)
Shrub (16%)	Shrub (16%)	Temperate broadleaf deciduous shrub (1%)	*Hippophae neurocarpa* shrub (1%)
		Subalpine broadleaf deciduous shrub (15%)	*Salix oritrepha* shrub (8%) *Salix oritrepha*,* Dasiphora fruticosa* shrub (1%) *Dasiphora fruticosa* shrub (5%) *Salix gilashanica* shrub (1%)
Barren land (20%)	Desert (1%)	Temperate semi‐shrubby and dwarf semi‐shrubby desert (1%)	*Sympegma regelii* desert (1%)
	Alpine vegetation (19%)	Alpine sparse vegetation (19%)	*Saussurea medusa*,* Saussurea* spp. sparse vegetation (15%) *Saussurea* spp. *Rhodiola rosea*,* Cremanthodium* spp. sparse vegetation (4%)
Water (3%)	Land without vegetation (3%)	Land without vegetation (3%)	River system (2%) Glaciers and snow limit (1%)

The environmental data used in this study comprised of 33 factors:

Topography: altitude, slope, aspect.

Soil: soil moisture measured at 2 cm from surface (SM2), soil moisture measured at 100 cm from surface (SM100), topsoil gravel content (GRAVEL), topsoil bulk density (BD), reference soil depth (RD), topsoil reference bulk density (RBD), topsoil organic carbon (OC), topsoil pH (PH), cation exchange capacity of the clay fraction in the topsoil (CEC), topsoil base saturation (BS), topsoil salinity (ECE), soil texture (texture), frozen soil (FS).

Precipitation: mean annual precipitation (MAP), growing‐season precipitation (GSP), actual evapotranspiration (AET), groundwater depth (ZWT), potential evapotranspiration (PET).

Temperature: active accumulated temperature (≥0°C) (AAT0), active accumulated temperature (≥10°C) (AAT10), mean annual temperature (MAT), growing‐season temperature (GST), mean temperature of the coldest month (MTCO), mean temperature of the warmest month (MTWA), mean annual bio‐temperature (MAB).

Light and radiation: solar radiation (RAD), downward atmospheric long‐wave radiation (L d), surface pressure (PSFC), annual average insolation duration (AST).

Human disturbance: Calculated by affected areas of cultivated fields, residential and roads.

Topographical data (ASTER GDEM V2, spatial resolution of 30 m) were provided by Data Cloud of Chinese Academy of Sciences (http://www.csdb.cn/). Farmland data were interpreted from Landsat8 OLI (http://glovis.usgs.gov/). The potential evapotranspiration ratio and MAB were calculated by the method provided by Holdridge (1967), and other data were provided by the Cold and Arid Regions Sciences Data Center at Lanzhou (http://westdc.westgis.ac.cn).

### 2.3 Data preparation

Attribute data, such as aspect, were changed into numeric data. Flat areas, shady slopes, semi‐shady slopes, semi‐sunny slopes, and sunny slopes were assigned to 1, 2, 3, 4, and 5, respectively. Nonsoil, sandy soil, loam, silty soil, clay loam, and clay were assigned to 1, 2, 3, 4, 5, and 6, respectively. Affected areas of cultivation activities, road traffic, and settlement were used to represent human activities. The measurement method was as follows: The 100‐m buffer for cultivated fields indicated the range of influence of cultivation activities. Buffers for city, county, town, and village residences were 500, 300, 100, and 50 m, respectively. Buffers for highway, national, province, county, town, and other roads were 1,000, 800, 500, 300, 100, and 50 m, respectively (Forman & Alexander, 1998; Liu et al., 2008). All buffers were then combined, and 1 and 0 were assigned to represent affected and unaffected areas.

### 2.4 Methods for the analysis of vegetation patterns

Seven vegetation patterns characteristics represent composition and configuration were calculated using version 4.2 of the freeware program Fragstats (McGarigal, 2002):

Three metrics represent composition, that is, Number of Patches (NP), Patch Richness (PR), and Mean Shape Index (SHAPE).

Four metrics represent configuration, that is, Largest Patch Index (LPI), Interspersion and Juxtaposition Index (IJI), Connectance Index (CONNECT), and Shannon's Diversity Index (SHDI).

The improved base vegetation map scale was 1:100,000, and the elementary patch area was about 250,000 m^2^, and 1 and 10 km were regarded as the minimal extent and the maximal extent for individual vegetation maps. The following areas were used to conduct points analysis: 1 × 1 km^2^, 2 × 2 km^2^, 3 × 3 km^2^, 5 × 5 km^2^, 7.5 × 7.5 km^2^, 10 × 10 km^2^, 12.5 × 12.5 km^2^, 15 × 15 km^2^, 20 × 20 km^2^, 25 × 25 km^2^, and 30 × 30 km^2^ (Peng et al., 2016; Wu, Shen, Sun, & Tueller, 2002). Following the calculation of landscape metrics from 1 × 1 km^2^ to 30 × 30 km^2^, the optional extent was selected, and the metrics were calculated using the optional extent (Du, Wang, & Guo, 2014; Wrbka, Schindler, Pollheimer, Schmitzberger, & Peterseil, 2008).

### 2.5 Correlation analysis

All landscape metrics and environment variables were submitted to the Shapiro–Wilk test for normality, a basic requirement for further application of parametric tests (Daniel, 1990), and all variables showed normal distribution.

The vegetation metrics and environmental data were analyzed using a direct ordination performed in the CANOCO for Windows program (version 4.5) (Braak & Smilauer, 2002; Hejcmanovā‐Nežerková & Hejcman, 2006) at different scales including LULC, vegetation groups, vegetation types, and formations and subformations.

Detrended correspondence analysis (DCA) was conducted for landscape metrics data to detect the length of the species gradient. After DCA, redundancy analysis (RDA) was used because the length of gradients was smaller than 3 (Lepš & Smilauer, 2003). The Monte Carlo permutation test was used to assess the significance of the canonical axes showing the relationship between landscape metrics and the selected environmental variables. Forward selection of variables (Escoufier & Roberts, 1979; Montgomery & Peck, 1982) was used to determine the relative importance of environmental variables in the input data, and the variance explained by them. The results of the RDA were plotted as two‐dimensional graphs using CANODRAW (Version 4.5). The continuous environmental variables were plotted as arrows originating from the centre of the graph. Correlation statistical analyses were performed by the forward selection analysis method using CANOCO for Windows 4.5.

## 3 RESULTS

### 3.1 Optional spatial extent analysis

**Figure 3:**
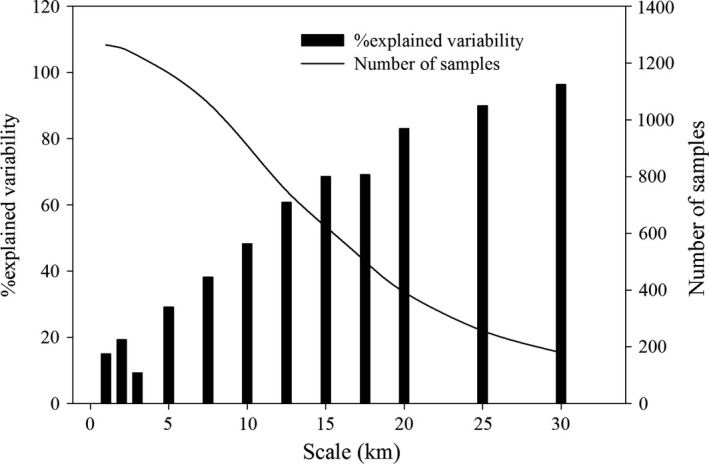
Explained variance, number of samples, and scale to choose the optional spatial extent

A series of area extent was used to calculate vegetation landscape metrics and to determine the optional spatial extent. The influence of spatial scale was found to be highly significant. Within these spatial extents, at 15 × 15 km^2^, explained variability was more than 60%; sample's number was more than half of total vegetation patches; and these samples were distributed all over the study area, 15 × 15 km^2^ being found to be the appropriate optional extent (Figure 3).

### 3.2 Correlation between landscape metrics and environment

At the LULC level, the most abundant land cover types were pasture (55%), barren land (20%), shrub (16%), and forest (5%) (Table 1). Forward selection of variables indicated that atmospheric counter radiation accounted for 19% variance (*p = *.002; *F = *118.81) and was higher than that of any other variables. The effect of soil moisture measured at 2 cm from surface and groundwater depth was significant and explained 6% (*p = *.002; *F = *43.78) and 5% (*p = *.002; *F = *27.86) of the variance, respectively (Table 2).

**Table 2:** Results of redundancy analyses at LULC level. Variables abbreviations: soil moisture measured at 2 cm from surface (SM2), soil moisture measured at 100 cm from surface (SM100), topsoil gravel content (GRAVEL), topsoil bulk density (BD), reference soil depth (RD), topsoil reference bulk density (RBD), topsoil organic carbon (OC), topsoil pH (PH), cat ion exchange capacity of the clay fraction in the topsoil (CEC), topsoil base saturation (BS), topsoil salinity (ECE), soil texture (Texture), frozen soil (FS); mean annual precipitation (MAP), growing‐season precipitation (GSP), actual evapotranspiration (AET), groundwater depth (ZWT), potential evapotranspiration (PET); active accumulated temperature (≥0°C) (AAT0), active accumulated temperature (≥10°C) (AAT10), mean annual temperature (MAT), growing‐season temperature (GST), mean temperature of the coldest month (MTCO), mean temperature of the warmest month (MTWA), mean annual bio‐temperature (MAB); solar radiation (RAD), atmospheric counter radiation (ACR), surface pressure (PSFC), annual average insolation duration (AST). The bold values represent that factors with such values could explain more than 5% of the variance

Category	Variable	Explained variance (%)	*p*‐value	*F*‐value
Topography	Altitude	2	.006	4.56
	Slope	2	.002	11.16
	Aspect	0.04	.948	0.24
Soil	Texture	0.07	.006	4.68
	SM2	**6**	.002	43.78
	SM100	3	.002	22.3
	OC	1	.002	6.19
	PH	0.07	.004	4.89
	ECE	1	.002	16.33
	CEC	0	.002	5.22
	RD	1	.002	5.98
	FS	0.01	.2	1.49
	BS	0.01	.07	2.18
	BD	1	.002	5.76
	RBD	1	.002	8.25
	GRAVEL	0.03	.776	0.5
Precipitation	MAP	2	.002	21.33
	GSP	1	.002	19.15
	ZWT	**5**	.002	27.86
	AET	1	.032	2.79
	PET	0.01	.026	3.59
Temperature	MAT	2	.002	18.12
	AAT0	0.01	.002	7.6
	AAT10	1	.002	10.15
	MTCO	0	.002	5.71
	MTWA	3	.002	27.46
	MAB	0.01	.988	0.12
	GST	1	.002	9
Light and radiation	RAD	2	.002	17.2
	ACR	**19**	.002	118.81
	AST	2	.002	19.39
	PSFC	1	.002	16.31
Human disturbances	Human	1	.002	6.24

The sum of all canonical eigenvalues was 0.59 (i.e., 59% of the total variance was explained, total variance equals 1.00). Eigenvalue of axes 1 was 0.334 and was 0.118 for axes 2. (i.e., 33.4% and 11.8% of the total variance was explained separately). Light and radiation, soil, temperature, precipitation, topography, and human disturbances accounted for 41%, 24%, 15%, 12%, 7%, and 1% of all the explanatory environmental variables, respectively (*p *≤* *.05) (Table 2).

**Figure 4:**
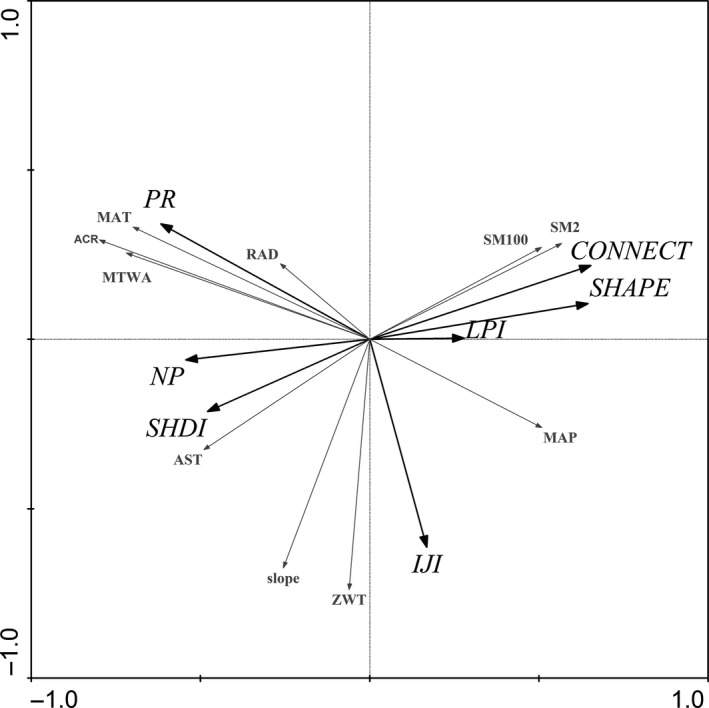
Redundancy analysis diagram in the upper reach of Heihe River basin with respect to landscape metrics and environmental factors of LULC. Gray arrows represent environmental factors, black arrows represent landscape metrics, Number of Patches (NP), Patch Richness (PR), Mean Shape Index (SHAPE), Largest Patch Index (LPI), Interspersion and Juxtaposition Index (IJI), Connectance Index (CONNECT) and Shannon's Diversity Index (SHDI). Other descriptions are same as Table 2

Patch Richness, Number of Patches, Connectance Index, Shannon's Diversity Index, and Mean Shape Index had the highest relation to atmospheric counter radiation, Interspersion and Juxtaposition Index had the highest relation to groundwater depth. Patch Richness, Number of Patches, Mean Shape Index, and Shannon's Diversity Index had positive correlation with mean annual temperature, mean temperature of the warmest month, soil moisture measured at 2 cm from surface, annual average insolation duration, respectively (Figure 4).

At the vegetation group level, the most abundant vegetation groups were meadow (43%), alpine vegetation (19%), shrub (16%), steppe (12%), and needleleaf forest (5%) (Table 1). Atmospheric counter radiation accounted for 16% of variance (*p *=* *.002; *F *=* *99.8) and was higher than that of any other variables. The effects of soil moisture measured at 2 cm was significant and explained 5% (*p *=* *.002; *F *=* *30.4) of the variance (Table 3).

**Table 3:** Results of redundancy analyses at vegetation group level. Abbreviations and meanings of bold values are same as Table 2

Category	Variable	Explained variance (%)	*p*‐value	*F*‐value
Topography	Altitude	2	.112	1.68
	Slope	1	.004	4.97
	Aspect	0.03	.946	0.23
Soil	Texture	0.01	.002	6.56
	SM2	**5**	.002	30.4
	SM100	4	.002	31.09
	OC	0.02	.008	4.13
	PH	1	.002	11.11
	ECE	2	.002	20.46
	CEC	1	.002	12.4
	RD	0.01	.044	2.4
	FS	1	.004	4.18
	BS	1	.004	5.04
	BD	1	.002	10.18
	RBD	0.04	.106	1.94
	GRAVEL	0.01	.448	0.96
Precipitation	MAP	2	.002	19.06
	GSP	1	.002	15
	ZWT	4	.002	32.11
	AET	0.01	.05	2.31
	PET	0.01	.126	1.69
Temperature	MAT	3	.002	24.14
	AAT0	1	.002	11.45
	AAT10	1	.002	9.93
	MTCO	1	.002	12.92
	MTWA	2	.002	8.73
	MAB	0.01	.85	0.36
	GST	1	.002	13.1
Light and radiation	RAD	3	.002	21.02
	ACR	**16**	.002	99.8
	AST	2	.002	18.21
	PSFC	3	.002	28.59
Human disturbances	Human	1	.002	9.38

The sum of all canonical eigenvalues was 0.60. Eigenvalue of axes 1 was 0.348, and was 0.11 for axes 2. Light and radiation, soil, temperature, precipitation, topography, and human disturbances accounted for 41%, 24%, 15%, 12%, 5%, and 1% of all the explanatory environmental variables, respectively (*p *≤* *.05) (Table 3).

**Figure 5:**
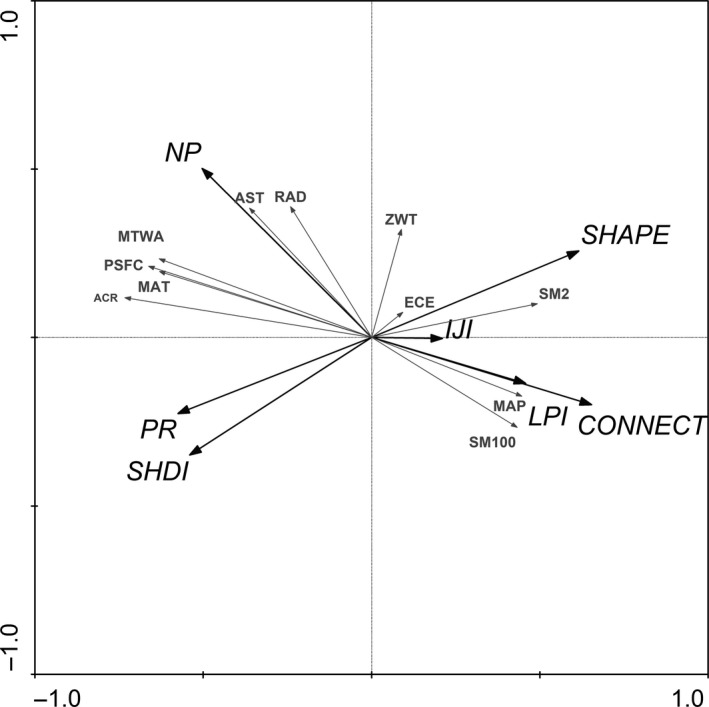
Redundancy analysis diagram in the upper reach of Heihe River basin with respect to landscape metrics and environmental factors of vegetation groups. Other descriptions are same as Table 2 and Figure 4

Patch Richness, Number of Patches, Connectance Index, Shannon's Diversity Index, and Mean Shape Index had the highest relation to atmospheric counter radiation. Number of Patches, Connectance Index, and Mean Shape Index had positive correlation with annual average insolation duration, soil moisture measured at 100 cm from surface, and soil moisture measured at 2 cm from surface, respectively. However, Largest Patch Index, Interspersion, and Juxtaposition Index were not significantly explained by environmental factors (Figure 5).

At the vegetation‐type level, the most abundant vegetation types were alpine *Kobresia* spp. and forb meadow (43%); alpine sparse vegetation (19%), subalpine broadleaf deciduous shrub (15%), temperate needlegrass arid steppe (6%), alpine grass, *Carex* steppe (5%), and cold–temperate and temperate mountain needle‐leaf forest (5%) (Table 1). Atmospheric counter radiation accounted for 19% of the variance (*p *=* *.002; *F *=* *126.15) and was higher than that of any other variables. The effect of soil moisture measured at 2 cm was significant and explained 7% (*p *=* *.002; *F *=* *46.37) of the variance (Table 4).

**Table 4:** Results of redundancy analysis at vegetation‐type level. Abbreviations and meanings of bold values are same as Table 2

Category	Variable	Explained variance (%)	*p*‐value	*F*‐value
Topography	Altitude	2	.244	1.24
	Slope	0.07	.002	7.41
	Aspect	0.02	.218	1.41
Soil	Texture	0.02	.002	5.13
	SM2	**7**	.002	46.37
	SM100	4	.002	26.02
	OC	0.01	.002	4.43
	PH	1	.002	9.18
	ECE	0.01	.222	1.37
	CEC	1	.002	8.4
	RD	0.01	.8	0.47
	FS	0.02	.012	3.08
	BS	1	.002	5.91
	BD	1	.002	12.03
	RBD	1	.002	7.94
	GRAVEL	0.03	.016	2.97
Precipitation	MAP	3	.002	11.62
	GSP	0.03	.002	16.36
	ZWT	3	.002	22.78
	AET	1	.012	4.17
	PET	0.01	.34	1.21
Temperature	MAT	2	.002	28.87
	AAT0	1	.002	7.29
	AAT10	1	.002	8.93
	MTCO	1	.004	5.28
	MTWA	1	.002	9.38
	MAB	0.01	.998	0.06
	GST	2	.002	17.29
Light and radiation	RAD	3	.002	21.5
	ACR	**19**	.002	126.15
	AST	2	.002	16.23
	PSFC	2	.002	20.94
Human disturbances	Human	1	.002	9.13

The sum of all canonical eigenvalues was 0.60. Eigenvalue of axes 1 was 0.364 and was 0.109 for axes 2. Light and radiation, soil, temperature, precipitation, topography, and human disturbances accounted for 44%, 27%, 13%, 12%, 3%, and 1% of all the explanatory environment variables, respectively (*p *≤* *.05)(Table 4).

**Figure 6:**
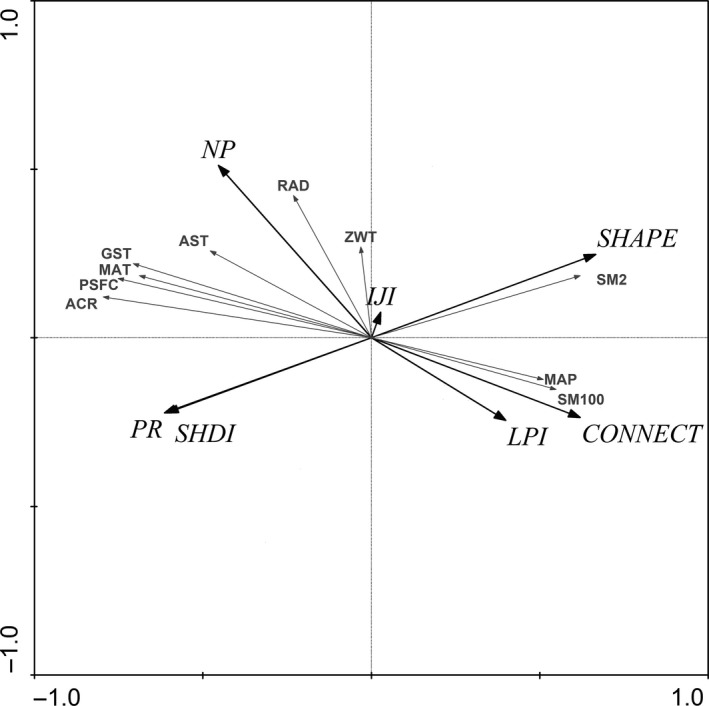
Redundancy analysis diagram in the upper reach of Heihe River basin with respect to landscape metrics and environmental factors of vegetation types. Other descriptions are same as Table 2 and Figure 4

Patch Richness, Number of Patches, Connectance Index, Shannon's Diversity Index, and Mean Shape Index had the highest relation to atmospheric counter radiation. Number of Patches, Connectance Index, and Largest patch index had correlation with solar radiation, soil moisture measured at 100 cm from surface, and annual average insolation duration, respectively. Mean Shape Index had positive correlation with soil moisture measured at 2 cm from surface. Interspersion and Juxtaposition Index was not significantly explained by environmental factors (Figure 6).

At the formation and subformation level, the most abundant vegetation formations and subformations were *Kobresia pygmaea* alpine meadow (26%), *Saussurea medusa* and *Saussurea* spp. sparse vegetation (15%), *Kobresia filifolia* alpine meadow (8%), *Salix oritrepha* shrub (8%), *Stipa purpurea* alpine steppe (6%), *Dasiphora fruticosa* shrub (5%), and *Picea crassifolia* forest (5%) (Table 1). Atmospheric counter radiation accounted for 24% of variance (*p *=* *.002; *F *=* *195.05) and was higher than that of any other variables. The effect of MAT, soil moisture measured at 2 cm, and soil moisture measured at 100 cm were significant and explained 8% (*p *=* *.002; *F *=* *75.4), 6% (*p *=* *.002; *F *=* *56.36), and 5% (*p* = .002; *F *=* *53.68) of the variance, respectively (Table 5).

**Table 5:** Results of redundancy analysis at vegetation formations and subformation level. Abbreviations and meanings of bold values are same as Table 2

Category	Variable	Explained variance (%)	*p*‐value	*F*‐value
Topography	Altitude	2	.01	4.31
	Slope	0.02	.186	1.61
	Aspect	0.01	.134	1.81
Soil	Texture	0.03	.002	8.45
	SM2	**6**	.002	56.36
	SM100	**5**	.002	53.68
	OC	0.01	.006	4.69
	PH	1	.002	5.73
	ECE	0.01	.63	0.72
	CEC	0.01	.006	4.28
	RD	0.02	.044	2.66
	FS	0.02	.002	5.21
	BS	0.01	.008	4.03
	BD	0.06	.002	8.13
	RBD	1	.004	4.36
	GRAVEL	1	.044	2.56
Precipitation	MAP	0.08	.002	12.75
	GSP	1	.002	4.16
	ZWT	2	.002	25.53
	AET	0.01	.232	1.44
	PET	0.01	.158	1.59
Temperature	MAT	**8**	.002	75.4
	AAT0	0.03	.002	17.64
	AAT10	2	.002	5.03
	MTCO	3	.002	37.4
	MTWA	1	.002	5.45
	MAB	0.01	.158	1.59
	GST	1	.002	8.37
Light and radiation	RAD	2	.002	27.84
	ACR	**24**	.002	195.05
	AST	2	.002	18.48
	PSFC	1	.002	19.45
Human disturbances	Human	0.01	.182	1.64

The sum of all canonical eigenvalues was 0.63. The eigenvalue of axes 1 was 0.449 and was 0.086 for axes 2. Light and radiation, Temperature, soil, precipitation, topography, and human disturbances accounted for 49%, 25%, 24%, 5%, 3%, and 0% of all the explanatory environmental variables, respectively (*p *≤* *.05) (Table 5).

**Figure 7:**
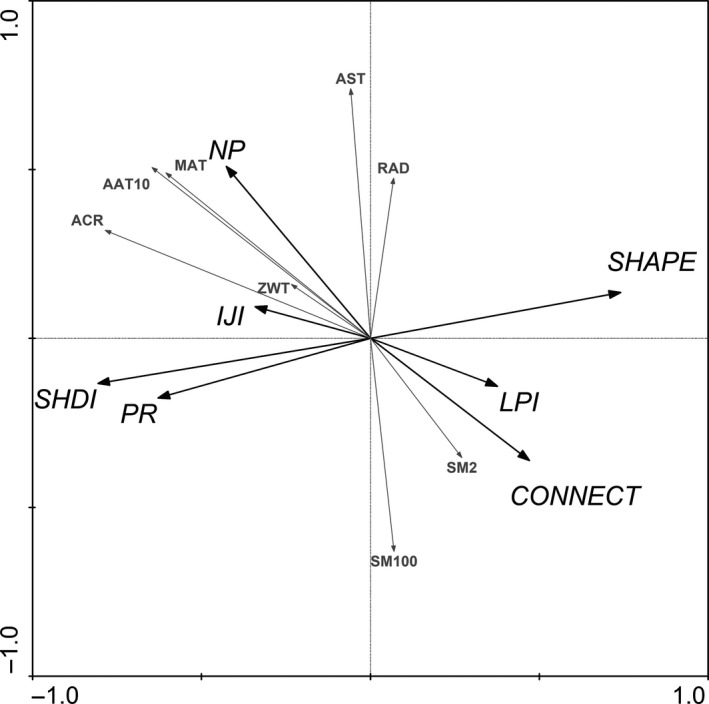
Redundancy analysis diagram in the upper reach of Heihe River basin with respect to landscape metrics and environmental factors of vegetation formations and subformations. Other descriptions are same as Table 2 and Figure 4

Patch Richness, Number of Patches, Connectance Index, Largest Patch Index, Interspersion and Juxtaposition Index, Shannon's Diversity Index, and Mean Shape Index had the highest relation to atmospheric counter radiation. Number of Patches and Connectance Index had correlation with mean annual temperature and soil moisture measured at 2 cm from surface, respectively (Figure 7).

## 4 DISCUSSION

### 4.1 Factors determining vegetation patterns at different levels

Different sets of environmental factors influenced vegetation patterns at different classification levels; this was also true in Heihe River basin.

1. Factors that could explain more than 5% of the vegetation pattern variance: Atmospheric counter radiation was the most important factor; it had the highest relation to Patch Richness, Number of Patches, Connectance Index, Largest Patch Index, Shannon's Diversity Index, and Mean Shape Index at the four levels. The fact that atmospheric counter radiation is directly related to the greenhouse effect and its relatively small year‐to‐year variation makes it one of the most promising parameters to monitor greenhouse effect‐related changes of the atmosphere and should be considered in climate research in mountain areas (Marty et al., 2003; Wild, Ohmura, & Cubasch, 1997).

Soil moisture is important for assessing water availability for plant growth in alpine prairies because it impacts nutrient uptake (He, Xing, & Bai, 2014). The distribution pattern of soil water is considered as the key factor to restrict the vegetation status (Xie et al., 2015). Although many soil factors were considered, only the effect of soil moisture measured at 2 and 100 cm from surface had significant effect on vegetation pattern in the upper reaches of Heihe River. Soil moisture measured at 2 cm from surface had significant relations to Mean Shape Index at levels except formation and subformation. Connectance Index was significantly explained by soil moisture measured at 2 cm from surface at the vegetation group level and by soil moisture measured at 100 cm from surface at the formation and subformation level, respectively.

2. Ordination of important environmental factors category: Many studies have shown that vegetation landscape composition and configuration is most affected by temperature in alpine areas (e.g., Chen, An, Inouye, & Schwartz, 2015). In the upper reach of Heihe River, light and radiation was the most important at the four levels. Soil had the second highest impact on the vegetation pattern except at formation and subformation level. Explained variance by temperature and precipitation was basically equal except at formation and subformation level. At formation and subformation level, temperature was the second important factor.

Human disturbance was less important in the study area, because the upper reaches of the Heihe River basin were protected by the Chinese Government, and human activity was weak (Wu, 2011), the farmland only 1% of the total area, and settlements and the road system were also limited.

3. The sum of all canonical eigenvalues of each level was similar but the specific aspects of vegetation patterns explained by environmental factors were different.

There are some reports that environmental factors affecting vegetation distribution are different at different levels (Dias & Melo, 2010; Kariyeva, Van Leeuwen, & Woodhouse, 2012), and that it is insufficient to use the same environmental factors to analyze the relationship between vegetation and environment and then to predict the effect of environment changes on vegetation; consideration of suitable environmental factors should be based on the specific vegetation level (Kariyeva et al., 2012). Our research findings indicated that this was also true in the Heihe River basin, and that it is important for policy makers and local government to make appropriate policies for environmental conservation.

### 4.2 Common environmental factors determining vegetation pattern at different levels

The upper reaches of the Heihe River basin are in an alpine region, which is part of the Tibetan Plateau. For the four levels, atmospheric counter radiation had the highest correlation with landscape metrics. Atmospheric counter radiation was much more important for vegetation than precipitation, soil, and altitude. Because most of the study area was located in high mountains, energy should be the most important variable for maintaining vegetation, as reported by other studies (Qiu, Zeng, Chen, Zhang, & Zhong, 2013; Sohoulande Djebou et al., 2015).

MAP played a significant role on vegetation in LULC, vegetation group, and vegetation‐type levels in the Heihe River basin. Some researchers (e.g., Huete, 2016) have reported similar results. However, at the finest level, vegetation formation and subformation, such as temperate needlegrass arid steppe (usually in the shade, off the assembly line in the mountains from 2,000 to 3,200 m), sparsely flowering *Stipa* (3,600–4,500 m on the slopes or the lower part of the sand ditch slope) and *Stipa breviflora* (many born in altitude 700–4,700 m on stony slopes, dry hillsides, or valley terraces), the vegetation response to precipitation was not obvious.

Generally, the number of variables whose relationship to vegetation reached significant levels was roughly equal, varying from 26 to 28; the number of the same variables for the four levels was 20.

The factors affecting vegetation were basically same at the vegetation group level and the vegetation‐type level and were sufficient to analyze the relationship between environmental and vegetation patterns at LULC, vegetation group, and vegetation formation and subformation level in mountainous regions, and vegetation‐type level could be ignored. This information should be helpful to us in projecting the future effect of environment on vegetation (John, 2011).

### 4.3 Uncertainty of relationship between environment and vegetation

It is not reasonable to expect environmental factors to explain all variations in vegetation, because other factors may affect it, such as the history of the vegetation and disturbance activities (Motzkin et al., 1999).

Furthermore, in the present study, data matching was also a limiting factor. For example, the spatial resolution of temperature and precipitation was 0.05°; the spatial resolution of DEM, aspect, and slope was 30 m; and, additionally, the study area was a high mountain. Mismatched data may have had a negative effect on our attempts to reach a reliable conclusion. This problem might be solved in the future with more accurate observations both on the ground and in remote sensing observations (Austin, 2013).

## CONFLICT OF INTEREST

None declared.
